# The Thickening Dilemma: A Rare Case of Idiopathic Hypertrophic Pachymeningitis Mimicking Granulomatosis With Polyangiitis

**DOI:** 10.7759/cureus.79094

**Published:** 2025-02-16

**Authors:** Jonathan R Forrest, Urmimala Chaudhuri, William R Jevnikar, Katelyn Booher, Joseph C LaPorta

**Affiliations:** 1 Medicine, Wright State University Boonshoft School of Medicine, Dayton, USA; 2 Internal Medicine, Wright State University, Dayton, USA; 3 Infectious Diseases, Wright State University Boonshoft School of Medicine, Dayton, USA; 4 Neurology and Rehabilitation Medicine, University of Cincinnati College of Medicine, Cincinnati, USA

**Keywords:** corticosteroid treatment, granulomatosis with polyangiitis (gpa), headache disorders, idiopathic hypertrophic cranial pachymeningitis, idiopathic hypertrophic pachymeningitis, igg4 disease, ihp, neurosarcoidosis, rituximab therapy, wegner’s granulomatosis

## Abstract

Idiopathic hypertrophic pachymeningitis (IHP) is a rare, chronic inflammatory disorder characterized by fibrotic thickening of the dura mater. The etiology of IHP is currently unknown; however, IHP often mimics other inflammatory conditions (causes of secondary hypertrophic pachymeningitis) including neurosarcoidosis, granulomatosis with polyangiitis (GPA), and IgG4-related disease. IHP manifests clinically with a spectrum of neurologic symptoms, including headache, paresthesia, cranial nerve (CN) palsies, and seizures. Here, we discuss the diagnosis and management of a patient presenting with multiple CN palsies following influenza B infection who was initially suspected to have GPA (due to positive cytoplasmic antineutrophil cytoplasmic antibody (c-ANCA), cranial polyneuropathies, and possible nasopharyngeal involvement) but was ultimately diagnosed with IHP which was evident on diagnostic imaging. The patient was managed with rituximab due to its efficacy in steroid-refractory pachymeningitis and as a precautionary for ANCA-associated disease, and corticosteroids.

A 41-year-old man with hypertension, chronic otitis media requiring myringotomy with tympanostomy tube placement, and mastoiditis requiring mastoidectomy presented with dysphagia, dysarthria, and left facial weakness over the course of 10 days following an influenza B infection. Despite initial treatment with corticosteroids for inflammation, the patient developed CN polyneuropathy (CN V, VII, X, XII). Positive c-ANCA, cranial polyneuropathies, and possible nasopharyngeal involvement led to primary suspicion of GPA, so corticosteroids were initiated which improved dysarthria and dysphagia. However, subsequent steroid taper led to severe headaches. MRI then revealed smooth dural thickening and enhancement consistent with pachymeningitis. The patient was diagnosed with IHP by exclusion of all other known etiologies and MRI findings. He was treated with intravenous methylprednisolone, followed by rituximab. Despite resolution of complex neurologic symptoms, including dysphagia and CN polyneuropathies, recurrent headaches necessitated several emergency department visits, where migraine cocktails and increased prednisone provided relief. He remains under neurology care for ongoing management. Although we are currently uncertain as to the exact underlying pathophysiology responsible for his recurrent headaches, the mechanisms we propose as possibilities involve a combination of corticosteroid withdrawal (as headaches often followed steroid taper) and sequelae of IHP itself (active and chronic inflammation of the dura). Furthermore, it is currently unknown as to whether his otolaryngologic history was contributory. The case highlights the diagnosis and management of a rare case of IHP in a situation where a patient with a significant otolaryngologic history experienced intractable neurologic symptoms following a viral infection. An extensive work-up was conducted to identify the source of presentation. The patient was managed with medications that proved to be safe and beneficial to the outcome of this patient.

## Introduction

Idiopathic hypertrophic pachymeningitis (IHP) is a rare, chronic inflammatory disorder characterized by fibrotic thickening of the dura mater due to unknown pathophysiology [1]. The prevalence of hypertrophic pachymeningitis (including both secondary and idiopathic) has been reported to be about 0.949 per 100,000, half of whom are idiopathic in nature [2]. Clinically, IHP manifests a spectrum of neurologic symptoms, including headache, weakness, paresthesia, cranial nerve palsies, and seizures [3]. Due to its variable presentation, IHP can mimic several other inflammatory conditions (causes of secondary hypertrophic pachymeningitis) such as neurosarcoidosis, granulomatosis with polyangiitis (GPA), and IgG4-related disease, which adds to the diagnostic complexity and often leads to misdiagnosis [3]. Here, we present a rare case of a patient who developed multiple cranial nerve (CN) palsies over 10 days and was initially suspected of having GPA due to positive cytoplasmic antineutrophil cytoplasmic antibody (c-ANCA), cranial polyneuropathies, and possible nasopharyngeal involvement but was ultimately diagnosed with IHP due to exclusion of all other known etiologies and treated with high-dose corticosteroids and rituximab leading to resolution of near-majority of neurologic symptoms. Our case was unique in that our patient developed GPA-mimicking IHP following an upper respiratory viral infection and extensive otolaryngologic history.

## Case presentation

A 41-year-old man with a history of hypertension, chronic otitis media requiring myringotomy with tympanostomy tube placement, and left-sided mastoiditis requiring mastoidectomy presented with gradual-onset dysphagia, dysarthria, and left facial paresis over the course of 10 days following an influenza B infection. Due to initial suspicion that his presentation was secondary to recurrent otitis media being experienced concurrently, the patient underwent bilateral tympanostomy four days later. His symptoms continued. The patient then developed mastoiditis one week following tympanostomy placement and subsequently underwent left intact canal tympanomastoidectomy, which did not result in neurological improvement. Due to persistent left facial paresis and dysphagia, as well as new-onset aural fullness and hearing loss, the patient was admitted for further evaluation by otolaryngology. 

During hospitalization, he received a new tympanostomy tube along with antibiotics and a four-day intravenous dexamethasone course at 24 mg daily for inflammation. Following treatment, the patient developed CN polyneuropathy affecting CN V, VII, X, and XII, which prompted an extensive workup. Aerobic/anaerobic bacterial cultures, fungal, and acid-fast bacilli stains, and cultures, all showed no growth. Due to diagnostic uncertainty and complexity of the case, the patient was then transferred to another academic institution for further rheumatologic and neuroimmunologic workup, which revealed mildly positive c-ANCA (1:40), and unremarkable antinuclear antibody (ANA), immunoglobulin G4 (IgG4), C3, and C4 levels. Further rheumatologic evaluation considered primarily GPA (due to positive serum c-ANCA, cranial polyneuropathies, and possible nasopharyngeal involvement) with inflammatory etiology, although post-viral cranial neuropathy and infection were also considered. Subsequent magnetic resonance imaging (MRI) with fast imaging employing steady-state acquisition demonstrated focal enhancement of the left facial nerve at the intracanalicular segment and left geniculate ganglion. This was interpreted as a separate inflammatory process (possibly post-viral cranial neuropathy), but as the leading diagnosis was GPA at the time, the patient was started on a five-day course of prednisone at 50 mg daily, which led to resolution of dysarthria and dysphagia.

**Figure 1:**
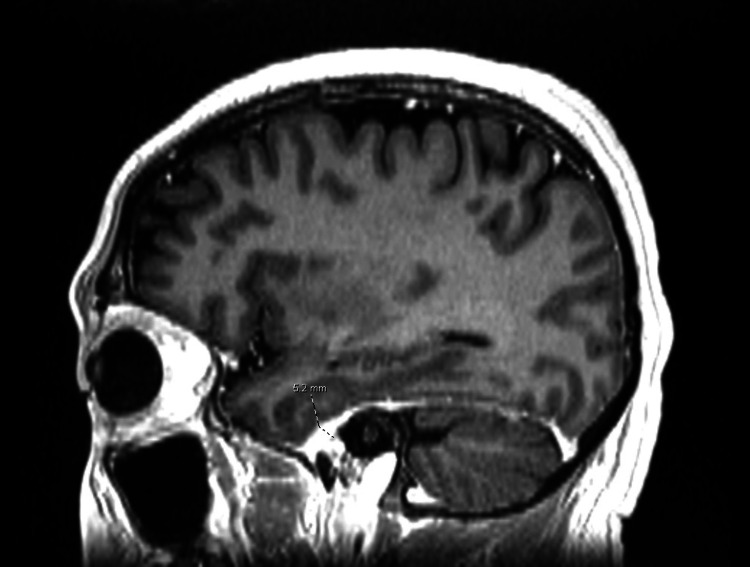
Sagittal T1-weighted MRI scan of the head showing 5.2 mm smooth dural thickening and enhancement anteriorly (dotted label) consistent with IHP, as opposed to GPA which typically displays local bone destruction at the base of the skull and vascular inflammation. MRI: Magnetic resonance imaging; IHP: Idiopathic hypertrophic pachymeningitis; GPA: Granulomatosis with polyangiitis

**Figure 2:**
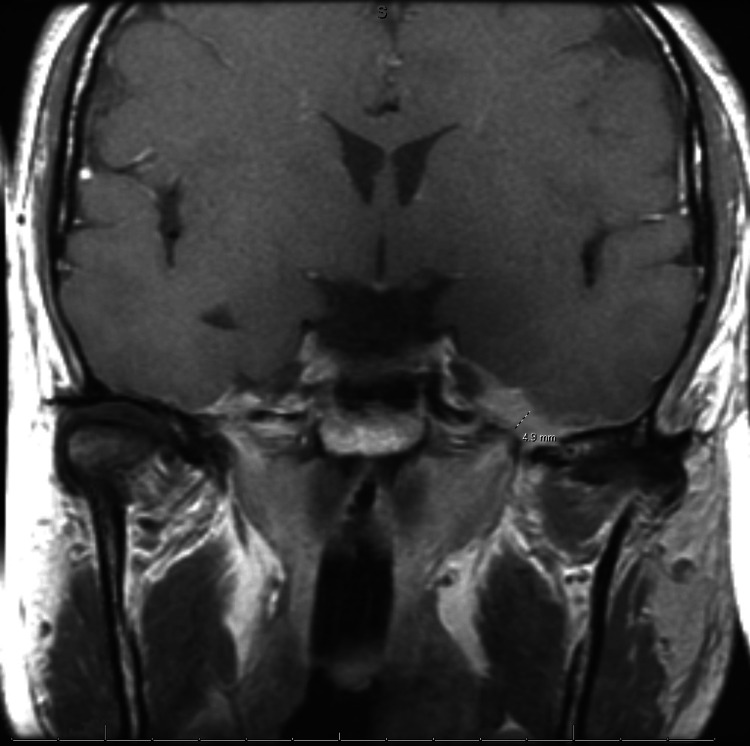
Coronal T1-weighted MRI scan showing 4.9 mm smooth dural thickening and enhancement (dotted label) consistent with IHP, as opposed to GPA which typically displays local bone destruction at the base of the skull and vascular inflammation. MRI: Magnetic resonance imaging; IHP: Idiopathic hypertrophic pachymeningitis; GPA: Granulomatosis with polyangiitis

Following steroid taper (prednisone 40 mg for seven days, followed by 30 mg for seven days, followed by 20 mg for 30 days), the patient developed severe headaches. Repeat MRI demonstrated ~5 millimeter smooth dural enhancement and thickening consistent with pachymeningitis (Figures 1, 2), and lumbar puncture findings included elevated oligoclonal bands and an elevated IgG index. Although these lumbar puncture findings were suspicious for IgG4-related disease, they were ultimately interpreted as non-specific inflammatory markers. IgG4-related disease was also ruled out due to negative initial serum IgG4 and MRI findings. Furthermore, although the patient did have clinically significant positive c-ANCA, diagnosis steered away from GPA due to a lack of other defining GPA clinical features such as renal and pulmonary vasculitis and imaging findings more characteristic of IHP. Positive c-ANCA, however, did support the diagnosis of IHP, as this finding is common in patients with IHP, and was thus interpreted as an atypical ANCA-associated inflammatory process. IHP was diagnosed by the neuroimmunologist, and he was treated with a five-day course of intravenous methylprednisolone at 1000 mg daily, followed by a prednisone taper (50 mg daily for 30 days with 10 mg reduction in dose every 30 days until 20 mg maintenance daily), resulting in symptomatic improvement. The clinical picture was most consistent with IHP, and rituximab was primarily considered and initiated (at 1000 mg on day 1 and day 15, with subsequent doses administered every six months as maintenance therapy) due to its first-line efficacy in steroid-refractory pachymeningitis and ANCA-associated disease, as well as CNS penetrance in inflammatory conditions.

Despite improvement with rituximab, the patient experienced recurrent headaches over the next six months, leading to three emergency department visits, where he was treated with migraine cocktails (consisting of intravenous saline, ketorolac, prochlorperazine, diphenhydramine, and magnesium sulfate) and increased prednisone dosing. His headaches resolved with this treatment. Apart from temporary increases in corticosteroid dosing and migraine cocktails for headaches, no modifications have been made to his long-term treatment regime, as the patient’s complex neurologic symptoms, including dysphagia and CN polyneuropathies, have resided. He continues to be monitored by neurology for ongoing management and will cease treatment once his clinical picture resolves entirely. Lack of resolution may warrant alternative treatment approaches.

## Discussion

IHP is a rare, chronic inflammatory disorder of unknown etiology, characterized by fibrotic thickening of the dura mater [1-3]. Clinically, IHP can present with a range of symptoms, including severe headaches, vision loss, weakness, paresthesia, CN palsies, and seizures [1,3]. While the exact pathogenesis remains unclear, IHP frequently mimics other inflammatory conditions (causes of secondary hypertrophic pachymeningitis) such as granulomatosis with polyangiitis (formerly known as Wegener’s granulomatosis), neurosarcoidosis, and IgG4-related disease [3-5]. Overlapping features between IHP and GPA include headache, positive serum ANCA (cytoplasmic antineutrophil cytoplasmic antibody) (either cytoplasmic or perinuclear), as well as cranial polyneuropathies [4]. Characteristics of both IHP and IgG4-related disease include increased dural thickness, non-specific inflammatory markers, and cranial neuropathies [4]. Features shared among both IHP and neurosarcoidosis include hearing loss, isolated CNS involvement, MRI showing dural thickening and enhancement, and relapse of neurologic symptoms following steroid taper [5]. Distinguishing features are depicted in Table 1. As characteristics of IHP and neurosarcoidosis are quite similar, it can be difficult to distinguish between them, often requiring dural biopsy, which remains the gold standard of diagnosis to exclude from other conditions [5]. Neurosarcoidosis was excluded in our patient due to the lack of features of systemic sarcoidosis (as cases of isolated neurosarcoidosis are <0.2 per 100,000). Additionally, our patient displayed positive serum ANCA which is found in about two-thirds of patients with IHP [4,5], whereas positive ANCA in neurosarcoidosis is much more rare. Furthermore, although dural thickening and enhancement may rarely be seen in neurosarcoidosis, it is the defining imaging manifestation and characteristic of IHP [6], which led us to our patient’s ultimate diagnosis. GPA was ruled out in our patient due to lack of pulmonary and renal vasculitis, as well as MRI findings more characteristic of IHP. MRI findings seen in those with GPA typically display local bone destruction at the base of the skull and vascular inflammation [6].

**Table 1:** Distinguishing features between IHP and mimicking (causes of secondary hypertrophic pachymeningitis) inflammatory conditions. Source: [4] IHP: Idiopathic hypertrophic pachymeningitis; ANCA: Antineutrophil cytoplasmic antibodies; ACE: Angiotensin-converting enzyme

	Neurological features	Specific laboratory findings or supportive diagnostic tests	Most commonly involved and characteristic organ involvement	Histologic findings on dural biopsy
Idiopathic hypertrophic pachymeningitis	Cranial polyneuropathies, cerebellar dysfunction	ANCA (weak association), non-specific inflammatory markers	N/A	Lymphoplasmacytic infiltrates with fibrous proliferation
Granulomatosis with polyangiitis	Cranial polyneuropathies, ophthalmoplegia, cerebrovascular events	ANCA	Lungs, kidney, nasopharynx	Necrotizing epithelioid granuloma
IgG4-related disease	Orbital pseudotumor, cranial polyneuropathies	Serum IgG4	Salivary gland, pancreas	Lymphoplasmacytic infiltrates with IgG4 cell predominance, storiform fibrosis, and obliterative phlebitis
Sarcoidosis	Facial nerve palsy, leptomeningitis, mass lesions	ACE, 1,25 OH D3	Lungs, hilar lymphadenopathy	Noncaseating epithelioid granuloma

Diagnosing IHP is challenging due to its low prevalence, varied clinical manifestations, and nonspecific imaging manifestations. Furthermore, dural or orbital tissue biopsy, a gold standard in diagnosis, carries inherent risks, such as hemorrhage, infection, and damage to nearby brain tissue [7,8], which is why this ultimately was not pursued in our patient. Additionally, based on the information we gathered as well as exclusion of all other known etiologies, we were able to make the diagnosis allowing us to defer dural biopsy. Diagnostic tools commonly employed include computed tomography, MRI, blood and cerebrospinal fluid analysis, general physical exam, and rheumatologic evaluation [8]. The presence of IHP may not be immediately apparent, underscoring the need for thorough and systematic evaluation.

The neurologic symptoms of IHP are primarily due to mass effect from the area of dural thickening, which results in CN, cortical, and cerebellar compression, as well as compression of cortical vessels resulting in inflammatory infiltration into the brain parenchyma [9]. Vascular compression by the lesion due to mass effect can precipitate venous congestion and local ischemia, leading to seizures, transient loss of consciousness, hemiparesis, and cognitive impairment [9]. The most common presenting symptom in IHP is headache, with studies indicating a correlation between the lesion location and the site of pain, as well as between the lesion size and pain severity [9,10]. CN involvement is the second most frequent manifestation, with CN II, III, IV, VI, and VII commonly affected [9,11]. Lesion-induced compression of the cerebellar or motor cortex may result in ataxia and involuntary movements, while compression of sensory nerves may cause numbness [11,12]. 

The management of IHP is primarily conservative, with corticosteroids being the first-line therapy [13]. Most patients require long-term corticosteroid treatment; however, prolonged steroid use can lead to significant adverse effects, including osteoporosis, hyperglycemia, hypertension, and increased susceptibility to infections [14]. Many patients do experience relapse of symptoms which is thought to be due to active and chronic inflammation of the dura [15]. For patients who are refractory to or intolerant of corticosteroids, second-line immunosuppressive agents such as rituximab, azathioprine, cyclophosphamide, and methotrexate are viable alternatives [16,17], although these have shown to increase risk of subsequent infection. Rituximab is known to be a good option for corticosteroid-refractory cases associated with ANCA-positive comorbidities, although studies suggest a larger population study may be required [17]. Our patient was managed with rituximab due to it being efficacious in corticosteroid-refractory pachymeningitis and as a precautionary for ANCA-associated disease. Despite the limited understanding of IHP pathophysiology, studies indicate that cyclophosphamide and mycophenolate mofetil may provide longer remission in corticosteroid-refractory cases [18]. In cases where pharmacologic interventions fail, surgical decompression has been shown to yield significant postoperative symptom relief and should be considered in patients with definite or progressive neurologic symptoms to prevent further deterioration [19,20]. The long-term course of hypertrophic pachymeningitis is known to follow one of three patterns: sustained remission, relapse with corticosteroid resistance, or relapse with corticosteroid dependence [21]. The exact timeline of symptom resolution is variable, however, among 96 patients with idiopathic hypertrophic spinal pachymeningitis, the recurrence rate has been documented to be 11% over a mean follow-up of one year and four months [21]. As many patients like ours do experience relapse, long-term follow-up is pivotal to determine the prognosis of IHP. These patients may require additional workup, including imaging, or modification to treatment strategies.

Further research is needed to determine contributions of otolaryngologic history to the development of IHP. However, there have been rare documented cases of pachymeningitis following chronic otitis media [22,23] as well as mastoiditis [23]. The pathogenesis of IHP from middle ear inflammation has been proposed to be mediated by venous return from emissary veins [22]. Our case differed, however, in that our patient’s symptoms persisted following bilateral tympanostomy and mastoidectomy, whereas the patients reported improvement after treatment of infection [22,23]. In patients with significant otolaryngologic and infectious history presenting with CN polyneuropathy, particularly in the presence of complications, IHP should remain on the differential. These patients should not only be aware of the possible complications that may arise, but also of the medical and surgical treatment options available. Although our patient did experience relapses of headaches over the course of six months during treatment with corticosteroids and rituximab, they were eventually able to achieve relief of complex neurologic symptoms, including dysphagia, dysarthria, and CN polyneuropathies, and have not needed further imaging.

## Conclusions

IHP is a rare, chronic inflammatory disorder characterized by fibrotic thickening of the dura mater and can be suspected in patients with significant otolaryngologic or infectious history presenting with CN polyneuropathy or intractable neurologic symptoms that do not resolve after treatment of underlying otolaryngologic infection. The purpose of presenting this case is to keep IHP on the differential diagnoses in patients with intractable neurologic symptoms such as headache, CN palsies, dysarthria, or vision changes, especially if there is an infectious or otolaryngologic history. Although there is not currently a well-established association between otolaryngologic or infectious pathology to IHP, we believe that our case may be representative of a possible association due to venous proximity. More research is needed to determine if this is the case. IHP should also be kept on the differential diagnoses in GPA-suspected patients (due to positive ANCA, which often leads to misdiagnosis) presenting with complex neurologic symptoms following an upper respiratory infection. Managing IHP is often by first-line corticosteroid therapy although there remains the possibility of long-term adverse effects. In steroid-refractory cases, second-line long-term immunosuppressants such as rituximab or cyclophosphamide are often considered. Our case proves that recognizing and medically treating IHP early on is beneficial symptomatically and to the patient’s health, although there remains the possibility of symptom (in our case, headache) relapse. Thus, it is crucial to continue to follow these patients’ health testimonies and pursue further imaging or workup if necessary.
